# Susceptibility to Ingroup Influence in Adolescents With Intellectual Disability: A Minimal Group Experiment on Social Judgment Making

**DOI:** 10.3389/fpsyg.2021.671910

**Published:** 2021-08-25

**Authors:** Sara Egger

**Affiliations:** Department of Special Needs Education, University of Fribourg, Fribourg, Switzerland

**Keywords:** peer influence, minimal group paradigm, social judgments, intellectual disability, adolescence, polarization

## Abstract

Adolescents with intellectual disability (ID) experience challenges and uncertainty when making judgments about other people’s intentions. In an attempt to achieve certainty, they might exhibit judgment tendencies that differ from those of typically developing adolescents. This study investigated social judgment making in adolescents with ID (*n* = 34, *M*_*age*_ = 14.89 years, *SD* = 1.41 years) compared with chronological age-matched adolescents without ID (*n* = 34, *M*_*age*_ = 14.68 years, *SD* = 1.15 years) and mental age (MA)-matched children (*n* = 34, *M*_*age*_ = 7.93 years, *SD* = 0.64 years). Participants used a computer-based task to judge the hostility of persons (fictitious characters). Adolescents with ID were found to make more polarizing judgments (i.e., either positive or negative, as opposed to moderate judgments) and were more likely to be guided by the opinions of a fictitious peer ingroup (minimal group) compared with adolescents without ID. No such differences were found between adolescents with ID and MA-matched children. The results are discussed in terms of scientific and practical implications.

## Introduction

Adolescents with intellectual disability (ID) often experience difficulties adequately judging the intentions of others, especially in ambiguous social situations and concerning hostile intentions (Leffert et al., 2010; Van Nieuwenhuijzen et al., 2011; Van Rest et al., 2020). Their limited cognitive and adaptive skills can lead them to trust other people, even when those people harbor hostile intentions (Greenspan et al., 2001, 2011; Snell et al., 2009; World Health Organization [WHO], 2019). Such difficulties in social judgment making can lead to uncertainty among adolescents with ID. Therefore, adolescents with ID may exhibit specific social judgment tendencies that result from them attempting to avoid ambiguity and reduce uncertainty. For instance, they may make more polarizing judgments (i.e., polarized positive or negative judgments, in contrast to moderate judgments; cf. Dhont and Hodson, 2014) or they may use social cues from their environment to feel more certain of their judgments (cf. Bybee and Zigler, 1998).

In order to better understand these processes, this study uses a computer-based task involving fictitious characters to assess the judgment tendencies of adolescents with ID, primary school mental age (MA)-matched children and adolescents without ID. The primary goal of this study is to investigate the extent to which adolescents with ID polarize their social judgments when judging hostile intentions. The results may provide an indication of individual judgment pattern preferences in adolescents with ID in circumstances in which no external references are available. However, social judgments are typically made in a social context in which various opinions conflict with one another. These different opinions are often represented by different social groups: groups who belong (i.e., ingroups) and others who do not (i.e., outgroups). Such intergroup behavior is common in the everyday lives of adolescents (e.g., during face-to-face conflicts in the schoolyard and also on social media; Berger, 2008; Müller et al., 2016; Wang and Edwards, 2016). A secondary goal of this study is therefore to investigate the extent to which adolescents with ID use cues from an ingroup of fictitious peers to inform their own social judgments, when cues from both an ingroup and an outgroup (i.e., minimal groups) are available.

### Polarization in Social Judgments

First impressions of a person are often made based only on minimal information. For example, clothing style or facial expressions are used to infer an unknown person’s hostility (Naumann et al., 2009; Zebrowitz, 2017; Over and Cook, 2018). In making social judgments based on minimal information, persons are influenced by their social experiences and affective state. For instance, adolescents with a history of antisocial behaviors have a tendency to perceive others as hostile, even if only minimal information is available and the judged persons display neutral facial expressions (Burt et al., 2009; Leist and Dadds, 2009).

Adolescents with ID exhibit specific judgment tendencies when judging unknown persons in ambiguous situations. For example, research indicates that children and adolescents with ID tend to encode more negative social information (Van Nieuwenhuijzen et al., 2004) and attribute more hostile intentions to others compared with their typically developing peers (Leffert et al., 2000, 2010; Van Nieuwenhuijzen et al., 2011; Van Rest et al., 2020). At the same time, adolescents with ID tend to judge the intentions of others uncritically and are therefore at risk of being manipulated by them (Greenspan et al., 2011; Buijs et al., 2017). Since adolescents with ID tend to over- or underestimate a person’s hostile characteristics in ambiguous social judgment situations, a polarizing judgment pattern in both directions can be expected among adolescents with ID. This type of polarizing judgment pattern for adolescents with ID has been observed in other contexts. For example, a questionnaire study about resistance to peer influence (Dekkers et al., 2017) and a previous experiment on social judgment both found a polarizing judgment pattern in adolescents with ID that was as pronounced as in younger children without ID (Kramer et al., 2009; Egger et al., 2021; but see Van Vaerenbergh and Thomas, 2013).

Polarization of social judgment among adolescents with ID can be explained in several ways. One is that adolescents with ID, like persons without ID (cf. Smith and Zárate, 1992), may draw on their pre-existing mental representations of other persons when making social judgments. These mental representations may be biased due to the specific social experiences of adolescents with ID (e.g., increased social conflict experiences; Allen, 2000; Douma et al., 2014). Biased mental representations (e.g., negative mental representations of persons) may therefore be more prevalent (cf. Hiemstra et al., 2019) among adolescents with ID faced with judging unfamiliar persons. Another explanation may refer to an often-assumed content-independent tendency of persons with ID to think in black-and-white terms (cf. Dekkers et al., 2017) and to the more general difficulties adolescents with ID and children without ID have incorporating subtle distinctions into their judgments (Fang et al., 2011; Mellor and Moore, 2014). An additional explanation may be a person’s content-independent preference for simplicity and an intolerance toward ambiguity and uncertainty during information processing (Naemi et al., 2009; Acar-Burkay et al., 2014), often referred to as a need for cognitive closure (cf. Webster and Kruglanski, 1994). Polarizing judgments, as opposed to moderate judgments, would accordingly reduce ambiguity and lead to a greater sense of certainty (Acar-Burkay et al., 2014). Although not yet investigated in adolescents with ID, studies among typically developing persons have demonstrated a correlation between a greater need for cognitive closure and characteristic attributes of persons with ID (e.g., high impulsivity and low mental flexibility; Webster and Kruglanski, 1994; Fujino et al., 2019). In conclusion, it is expected that in social judgment situations with minimal information more polarized social judgment making will be observed among adolescents with ID compared with adolescents without ID. This may be explained by reliance on biased mental representations, by a content-independent tendency toward a simplistic judgment style, by difficulties in differentiating judgments, or by a preference for unambiguous judgments (cf. Webster and Kruglanski, 1994; Fang et al., 2011; Dekkers et al., 2017; Hiemstra et al., 2019).

### Susceptibility to Peer Influence

In addition to individual tendencies, contextual influences can also affect the judgment behavior of adolescents with ID (Bybee and Zigler, 1998; Bexkens et al., 2019; Wagemaker et al., 2020). Judgments are often made in social contexts where the opinions of others can be influential (Sherman et al., 2016). The act of using other people’s opinions (e.g., peers’ opinions) to shape one’s own judgment is more pronounced among adolescents with ID than among typically developing adolescents (cf. Bybee and Zigler, 1998). This finding is evident in studies using self-reports on resistance to peer influence, experiments on risk-taking and on social judgment making regarding the coolness of photographed adolescents (Dekkers et al., 2017; Bexkens et al., 2019; Wagemaker et al., 2020; Authors). Uncertainty due to repeated experiences of failure to cope with everyday situations (e.g., social judgment situations) has been noted as a motive for heightened openness to social influences in adolescents with ID (Lustman and Zigler, 1982; Bybee and Zigler, 1998). This explanation derives specifically from the tradition of Outerdirectedness research, where task ambiguity is an important criterion and can increase uncertainty (cf. Bybee and Zigler, 1998). Like adolescents with ID, younger children without ID also exhibit a strong openness to peer influence (Lustman and Zigler, 1982; Bybee and Zigler, 1998). However, this openness may be due more to their general use of external cues and less to an age-specific openness to peer influence (cf. Egger et al., 2021). In particular, resistance to peer influence in neutral situations (in contrast to antisocial peer influence) decreases with age from childhood to early adulthood in typically developing persons (cf. Sumter et al., 2009). Although few studies exist that partially also include small sample sizes, the existing evidence suggests an increased openness to peer influence for adolescents with ID and younger MA-matched children (Bybee and Zigler, 1998; Bexkens et al., 2019; Wagemaker et al., 2020).

### Ingroup Favoritism

In everyday life, different subgroups of peers may influence the social judgment making of adolescents with ID. The few existing studies on this subject, mostly conducted in the 1960s and 1970s, suggest that adolescents with ID orient themselves toward different reference groups to varying degrees (cf. Stamm and Gardner, 1969; Strichart, 1974). Such studies examined, for example, the degree of orientation to peers from regular schools compared with peers from special school classes (Stamm and Gardner, 1969). They also analyzed the imitation of competent vs. non-competent peers and the orientation with liked vs. disliked peers (Strichart, 1974). To date, no studies regarding openness to ingroup influence in an intergroup context with an outgroup have been conducted with adolescents with ID. Therefore, an outstanding question is to what extent adolescents with ID orient toward opinions of an ingroup when an in- and outgroup of peers are present as potential reference groups (i.e., preference of an in- over an outgroup).

The extent to which a person favors an ingroup over an outgroup depends on several individual-level characteristics. One characteristic is age: A tendency toward ingroup favoritism begins around the age of 5 years (Dunham et al., 2011). Ingroup favoritism manifests itself in increased and generalized ingroup orientation around an age of 7 and becomes more nuanced depending on situational conditions by the age of 9 (Nesdale et al., 2005). This age-specific generalized ingroup favoritism and ingroup orientation can be explained with the low self-regulatory abilities up to an age of 9 (cf. ibid.). For example, Bigler et al. (1997) showed that in elementary school children group membership based only on assigned T-shirt color resulted in more positive ratings for the ingroup compared with the outgroup. Given their problems with self-regulation, adolescents with ID may also tend to have an increased generalized ingroup orientation (Danielsson et al., 2012; Bexkens et al., 2019). However, it should be noted that typically developing adolescents also orient toward ingroups of peers, particularly during early adolescence (Teichman et al., 2007; Tanti et al., 2011). This finding is explained by an age-specific increased desire to belong. Because adolescents with ID tend to have a limited circle of friends and a strong need to belong (cf. Strnadovà et al., 2018), they may show an even increased ingroup orientation compared with more socially embedded adolescents without ID. Furthermore, Tanti et al. (2011) demonstrated that adolescents with reduced cognitive abilities tended to favor an ingroup over an outgroup. In addition, people with a preference for unambiguous and simple explanatory patterns (i.e., need for cognitive closure) exhibit increased ingroup favoritism and outgroup derogation (i.e., low acceptance of outgroup attitudes). This ingroup favoritism therefore appears to satisfy a heightened need for social correctness and desire for certainty about one’s own attitudes and can lead to social verification with likeminded people (i.e., with an ingroup; Hogg and Abrams, 1993; Shah et al., 1998). Adolescents with ID, particularly those with comparatively higher IQs (i.e., mild ID), may be highly susceptible to the influence of an ingroup of peers; they are often aware of their own cognitive limitations and therefore frequently strive not to deviate negatively (Snell et al., 2009).

In summary, one expects that adolescents with ID are more strongly oriented toward an ingroup as opposed to an outgroup compared with adolescents without ID because of the former’s difficulties with self-regulation, cognitive limitations, increased need to belong and striving for social correctness. Children may also be strongly oriented to an ingroup of peers, as opposed to an outgroup, regardless of situational circumstances because of their reduced self-regulatory abilities.

### The Current Study

The present study uses an experimental research design to test the polarization of social judgment and the susceptibility to ingroup influence during social judgment making in adolescents with ID. The approach builds on a previous study with different experimental material on individual and contextual factors that influence social judgment making among adolescents with ID (Egger et al., 2021). Egger et al. (2021) found that adolescents with ID tended to be more polarizing in judging the social attractiveness (i.e., *coolness*) of photographed peers and were more open to peer influence than typically developing adolescents; they performed at levels comparable to children matched for MA (i.e., with a similar cognitive developmental level as the adolescents with ID; see also Weisz and Yeates, 1981). In the current study, the focus of social judgment is newly directed toward perceptions of hostility. This focus was chosen because of the likely difficulties and bias in hostility judgments experienced by adolescents with ID (Greenspan et al., 2011; Van Rest et al., 2020). Unlike the previous study, peers were no longer considered a homogenous group in this investigation but were instead differentiated into an in- and outgroup, in accordance with the minimal group paradigm (Diehl, 1990). So-called minimal group experiments refer to ingroups and outgroups that differ only on the basis of a trivial criterion, such as T-shirt color (cf. Moghaddam and Stringer, 1986; Diehl, 1990; MacDonald et al., 2013; Wilks et al., 2018).

Taken together, in the present study an experimental task on (1) polarizing judgment tendencies, and on (2) susceptibility to ingroup influence (ingroup bias) was developed. The goal of this study is to compare these judgment tendencies of adolescents with ID [experimental group (EG)] with adolescents without ID [comparison group 1 (CG1)]. In addition, this study includes a comparison group of MA-matched children [comparison group 2 (CG2)] to shed more light on the role of MA in these processes (cf. Bybee and Zigler, 1998).

**FIGURE 1:**
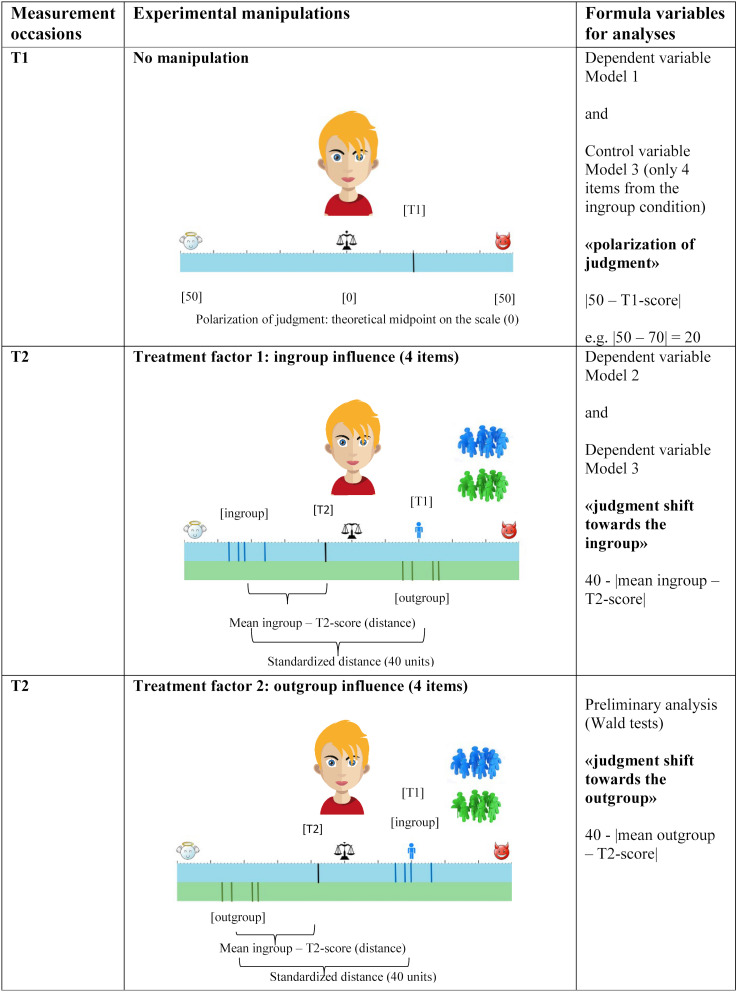
Overview on the experiment over the two measurements occasions. Picture source: Freepik (2020).

In the present study, participants were asked to complete a computer-based task in one session. They were shown images of unknown people (see Figure 1) and asked to rate their hostility under two experimental conditions (see Figure 1). For the first measurement (T1), no social influences were implemented. These ratings at T1 were used to analyze the degree of polarization in participant social judgments. In the second measurement (T2; same session as T1), participants were made aware of how a fictitious in- and outgroup (minimal groups) had rated each image, and they were then asked to rate each image again. Based on the theoretical reasoning described above, this study was primarily interested in influence from an ingroup. The outgroup condition only served as a comparison condition to estimate the ingroup’s influence. Participant ratings at T2 (see Figure 1, T2, treatment factor 1) were used to examine their susceptibility to ingroup influence. Consistent with the expectations outlined above, the following hypotheses were formulated.

Hypothesis 1: Adolescents with ID make more polarizing social judgments than adolescents without ID.

Hypothesis 2: Adolescents with ID are more open to ingroup influence than adolescents without ID.

In accordance with earlier findings (Egger et al., 2021), similar performance in polarizing social judgments and in susceptibility to ingroup influence was expected between adolescents with ID and MA-matched children.

Explorative analyzes were used to consider the role of different polarization tendencies as an explanation for the group differences in susceptibility to ingroup influence. In this regard, theoretical approaches point to a preference for a simplifying thinking style (i.e., the need for cognitive closure) associated with both polarized judgment making and increased ingroup bias (Shah et al., 1998; Naemi et al., 2009; Acar-Burkay et al., 2014).

## Materials and Methods

### Participants

This study was conducted within a larger research project related to peer influence and peer relations in individuals with ID (cf. Müller et al., 2020). The institutional research commission of the Department of Special Needs Education at the University of Fribourg reviewed and accepted the scientific procedures and ethical conduct of this study. Consent was obtained from the parents or legal guardians of each participant and from all participants prior to the study. Participants were given the opportunity to withdraw their participation at any time throughout the study.

Adolescents with ID were recruited at Swiss special needs schools for students with ID. Participants of the comparison groups attended regular schools. Overall, informed consent was obtained from a total of 324 participants (71 adolescents from special needs schools, 153 adolescents from regular schools and 100 children from regular schools). Due to technical problems and participant absences (e.g., due to illness) data were collected for 300 individuals (71 adolescents from special needs schools, 142 adolescents from regular schools and 87 children from regular schools).

Swiss special needs schools can only be attended by students who have a clinical diagnosis of ID as defined by the ICD-10 code, have been assessed using an IQ test (IQ < 70) and typically have received a clinical rating of adaptive behavior (World Health Organization [WHO], 2019). Therefore, the students enrolled in special needs schools that participated in this study all had a diagnosis of ID. In order to facilitate optimal group matching, an additional intelligence measure was collected using the short version of the Culture Fair Test-20R (CFT-20R; Weiss, 2006). Since best practices recommend acknowledging the Flynn effect and confidence intervals when obtaining an IQ score (Schalock et al., 2010), only students with an IQ of 75 or lower and with low adaptive competences compared with the reference norm were included. Adaptive competences were measured using a German version of the Adaptive Behavior Assessment System 3-teacher questionnaire (ABAS-3; Harrison and Oakland, 2015; Bienstein et al., 2017). The standardized *General Adaptive Composite* (GAC) value was calculated over all items in the social, practical and conceptual domains. The corresponding values can be differentiated into *very low* (≤70), *low* (71–79), *below average* (80–89), *average* (90–109), *above average* (110–119), and *high* (≥ 120). Students were included in the EG if they demonstrated adaptive behavior ranging from *very low* to *below average.* Because this study focuses on early- to mid-adolescence, only individuals between 12 and 17 years of age were included in the EG. In order to recruit EG participants, teachers at special needs schools were asked to distribute letters to parents of adolescents who were able to express themselves well verbally, had a good comprehension of language, could write their own name, and had a mild ID (no further specification). Of the 71 participants from special needs schools, 34 satisfied the study’s inclusion criteria for IQ (*n* = 11: IQ < 54, *n* = 15: IQ 54–69, *n* = 8: IQ 70–75), adaptive behavior (standardized GAC: *M* = 76.09, *SD* = 9.62, range = 51–89), and age (*M* = 14.89 years, *SD* = 1.38 years, range = 12.3–17.2 years). Because the CFT-20R norms provide no differentiation of IQ scores below 54, no specific IQ scores could be specified for the 11 participants with an IQ below 54. It is important to note, however, that these 11 participants had GAC scores (*M* = 69.45; *SD* = 10.38; range = 51–82) ranging from *very low* to *below average*, just like the other participants. Using observation protocols during the experimental task, no apparent motor or sensory deficits were observed and all participants were able to meet the technical requirements of the task.

Next, a pairwise matching procedure was conducted to identify participants for CG1 and CG2. To select the 34 participants of CG1 (IQ: *M* = 100.12, *SD* = 9.26), age and gender were used as the selection criteria. If no same-gender person with an age difference of up to 0.5 years was available, the next person with the smallest age difference, regardless of gender, was included in the sample. For the selection of the 34 children in CG2, matching was based on the CFT-20R raw score. If several participants had the same CFT-20R raw score, the person with the same gender was selected. Based on the relatively small number of potential children with the same MA for CG2 compared with the adolescents without ID for CG1, the priority in CG2 matching was the CFT-20R raw score but gender was still considered. The CFT-20R does not provide IQ scores for children of this age. Observation protocols indicated that the children in CG2 had no significant difficulties with the tasks or technical facilities.

Cross-tab tests revealed no significant gender differences between the groups (EG vs. CG1, *p* = 0.625; EG vs. CG2, *p* = 0.331; CG1 vs. CG2, *p* = 0.628). Consistent with the purpose of matching, the EG and CG1 did not differ significantly in chronological age (*p* = 0.522; see Table 1). As intended, participants in the CG2 were significantly younger in chronological age than participants in the EG (*p* < 0.001) and CG1 (*p* < 0.001; see Table 1). In terms of intellectual abilities (CFT-20R raw score), participants in CG1 had significantly higher CFT-20R raw scores than those in the EG (*p* < 0.001) and CG2 (*p* < 0.001; see Table 1), as expected. Finally, the participants in the EG and CG2 did not differ significantly in terms of CFT-20R raw scores (*p* = 0.051; see Table 1).

**TABLE 1:** Participant characteristics of each group.

	**EG (*N* = 34)**	**CG1 (*N* = 34)**	**CG2 (*N* = 34)**
Mean age in years (SD)	14.89 (1.41)_a_	14.68 (1.15)_a, b_	7.93 (0.64)_b_
Mean CFT-20R (SD)	18.88 (5.39)_a_	39.26 (4.12)_a, b_	21.53 (3.27)_b_
% Male	58.8	52.9	47.1

*Similar subscript letters refer to significant differences in means between groups, *p* < 0.001.*

*EG, experimental group comprising adolescents with intellectual disability (ID); CG1, comparison group 1 comprising chronological age-matched adolescents without ID; CG2, comparison group 2 comprising younger mental age-matched children.*

### Experimental Material

The computer-based task was programed using E-Prime software (Psychology Software Tools, 2016) and was developed by Authors. A 2 × 3 factorial design with a two-level within-subjects factor (no influence from ingroup vs. influence from ingroup) and a three-level between-subjects factor (EG vs. CG1 vs. CG2) was used. Research assistants administered the task at each school using a standardized script. Data from MA-matched children and adolescents without ID were collected in small group settings in quiet rooms (physical barriers were erected between desks to prevent students from looking at one another and from becoming distracted). Adolescents with ID performed the task in individual settings so that they could receive better support when they had questions. All participants received a 17.5-inch laptop with a touch screen and headphones for receiving standardized instructions.

#### Introduction of the Experimental Task

The research assistants informed the participants that they were taking part in a task about film characters and that these characters had been very carefully selected for use in movies. This framing was implemented to focus the participants’ attention on people being evaluated and to provide thematic embedding (Myers and Hansen, 2012). The parallels between virtually simulated images of people and real people were made clear by pointing out that characters in films often behave like real people and may therefore be nice or hostile.

In this task, participants were asked to rate the hostility of the pretend film characters. Images of fictitious virtually simulated people were used for the ratings. An advantage of using virtually simulated instead of actual people is the ability to create standardized characters that can be systematically manipulated in certain aspects. An identical section (upper body and facial expression) of each virtually simulated person was displayed in each image; the images differed only in gender, hair style and hair color, eye color and clothing (see the examples in the Supplementary Appendix). The gender of the virtually simulated persons was equally distributed over all of the rated images. Participants were not given any additional information about the characteristics of the virtually simulated people. Consistent with principles used in Outerdirectedness research (cf. Bybee and Zigler, 1998), these conditions ensured exposure to an ambiguous situation without obviously correct answers for all participants and allowed for the investigation of participants’ judgment style in such situations. All images were taken from a picture database^1^ and were graphically adapted to the task.

Each of the participants sat at a laptop and was told through headphones that they would rate the perceived hostility of each person depicted in images. Hostility was defined for participants as “lying, teasing, or telling nasty things about others.” The scale for judging hostility took the form of a bar presented below the pictures (width = 100 units, 9.3 inches, with symbols from right to left for *very hostile*, *medium hostile*, and *not hostile* above the bar; see Figure 1; Authors for a similar scale). An animated illustration was used to show participants that they could selected a value anywhere on the continuous scale (i.e., an animated finger pointed at different places on the scale). To introduce participants to the mechanism for rating each image, the scale was introduced systematically with exercises in which participants tapped certain areas on the scale (for example, showing the location of *very hostile*). Only after these exercises were completed correctly did the experimental procedure continue.

Participants selected their ingroup of peers by tapping one of two schematic peer group pictures. The only distinguishing feature between the in- and outgroup was their color assignment (blue or green; see Figure 1; T2). According to the minimal-group paradigm, differentiation based on color criterion is a possible minimal distinguishing feature of groups (e.g., group-specific T-shirt color), which can lead to stronger identification with the ingroup compared with the outgroup despite the low significance of the group characteristic (Bigler et al., 1997; Kinzler et al., 2010). Therefore, the influence of an ingroup can be examined on the sole basis of group membership. After group selection and an exercise that assessed the participants’ correct understanding of their group membership, the exact procedure of the task was explained step-by-step.

#### Main Task

The main task included two measurement points in the same session, the first without external manipulation and the second with an external manipulation. At the first measurement (T1), participants rated the hostility of the virtually simulated persons on a scale that had the same color as the own group (e.g., blue scale if blue group membership was selected; see Figure 1); a mark appeared on the scale to reflect their own rating without external manipulation. The distance between the participant’s mark and the middle of the scale (0–50) was used to measure the degree of polarization of participants’ social judgment (see Figure 1; T1). Higher values indicated more polarization of social judgments. At the second measurement (see Figure 1; T2), the participants’ own judgment was removed and a small figure appeared just above the scale to remind the participants of their original judgment at T1. Additionally, four ratings by members of the ingroup (e.g., colored blue) appeared on the same scale and four ratings by members of the outgroup appeared on an extended scale (e.g., colored green; see Figure 1; T2). The ingroup and outgroup were presented as two groups with closely aligned ratings (width: 10 units on the scale; see Figure 1; T2) in order to create the perception that the two groups had a homogeneous group norm. Homogeneous group opinions increase the pressure on group members to conform to their opinions to align with that of the group (cf. Hogg and Adelman, 2013). The positioning of the ingroup’s and outgroup’s ratings on the scale was based on defined criteria (see technical description below), with the own first rating having either the same position as the outgroup’s ratings (Figure 1; T2; treatment factor 1) or as the ingroup’s ratings (Figure 1; T2; treatment factor 2). These different arrangements of the ingroup’s and outgroup’s ratings (treatment factor 1 and 2) were presented in a randomized order to the participants. In order to measure susceptibility to external influences (e.g., ingroup influence in comparison to outgroup influence), participants were asked to mark their final rating on their scale (e.g., blue scale if blue group membership was selected; see Figure 1; T2). The task was completed only after all of the items were rated. This procedure ensured that no data were missing.

#### Technical Description of the Experimental Manipulation

The position of the in- and outgroup’s ratings (T2) were dependent on participants’ first ratings (T1). To investigate ingroup orientation, at T2 half of the items (four items) were given an ingroup rating that was placed at a standardized distance (i.e., ingroup mean at a distance of 40 units on the scale = 3.72 inches) from the participants’ first rating at T1 (see Figure 1; T2; treatment factor 1: ingroup influence). For T1 ratings between 0 and 50 on the scale (i.e., the left part of the scale), the position of the mean of the ingroup ratings (treatment factor 1) was set at a standardized distance on the right side of the initial rating. For T1 ratings above 50 (i.e., the right part of the scale), the mean of the ingroup ratings (treatment factor 1) was placed at a standardized distance on the left side of the participant’s first rating. At the same time, the mean of the outgroup rating was at the same position on the scale as participants’ rating at T1 (see ibid.). For ease of readability, the term “ingroup influence” will be used below to refer to ingroup influence considered in an intergroup context with an outgroup present.

The measured distance between the participant’s rating (T2) and the mean of the ingroup ratings was used to indicate susceptibility to ingroup influence (Figure 1; T2; treatment factor 1). Accordingly, a shorter distance implied a stronger orientation toward the ingroup. These measurements were used to conduct the primary analysis (Hypothesis 2). In contrast, at T1 the ratings of all participants were at exactly the same standardized distance from the mean of the ingroup ratings at T2 and therefore irrelevant for testing Hypothesis 2 (because there was no variability between individuals). Conversely, for the other half of the items (four items), the positions of the ingroup ratings and outgroup ratings were reversed on the scale (see Figure 1; T2; treatment factor 2: outgroup influence). This second group arrangement served as a comparison condition to for ingroup influence but was not included in the hypothesis tests.

To avoid the possibility that participants might recognize that the ingroup and outgroup ratings corresponded to their individual ratings at T1, additional distractor pictures (six additional pictures of virtually simulated people) were included. In these distractors, the judgments of both the ingroup and outgroup at T2 either corresponded to the participants’ judgment at T1 (3x) or varied across the entire scale (3x). Accordingly, participants rated a total of 14 virtually simulated persons at two time points.

After conducting the task, a debriefing session with the participants was conducted in which the tasks were explained and the topics of social judgment and external orientation in daily life were discussed.

### Statistical Analyses

Multilevel analyses were conducted taking into account the nested nature of the data: Participants (level 2) rated multiple experimental stimuli (level 1; cf. Smolik, 2010). The conventionally used aggregation of mean values in single-level analyses cannot account for intra-individual variability of individuals’ responses and therefore ignores the reliability differences of the aggregated means between participants (Nezlek, 2008; Smolik, 2010). Especially for individuals with a high intra-individual variability across different items of an experimental task, as is often the case with children (cf. Siegler, 1994) and in adolescents with ID, aggregated means might not be an accurate indicator (Nezlek, 2008). By implementing two levels (level 1: experimental stimuli, level 2: individuals; see below), one takes into account that different individuals might exhibit different levels of intra-individual variability in their response behavior to experimental stimuli (residuals on level 1 and 2). Therefore, more accurate group means can be estimated since the coefficients on level 2 are weighted by the reliability of the measured scores on level 1 (ibid.). Multilevel analyses were performed using Mplus 8.1 software (Muthén and Muthén, 2017).

In preliminary analyses, intraclass correlations (ICCs) were calculated using the software SPSS to examine the structure of the data. Next, the data were tested for important statistical assumptions with respect to the primary analyses (homogeneity of variances, a normal distribution and outliers).

To test Hypothesis 1 regarding group differences in judgment polarization, a means-as-outcomes model was used in accordance with Luke (2004). This model allowed participants’ variability in responses at level 1 (experimental stimuli) to be taken into account (cf. Geiser, 2011). It was tested whether differences between the clusters (individuals) in the dependent variable (polarization of social judgment) were predicted by the level 2 predictor group membership (EG as a reference category, dummy coded; cf. Geiser, 2011). The variable *polarization of social judgment* was measured at T1 for all items (see Figure 1).

In order to assess the role of ingroup vs. outgroup orientation, Wald tests (Bühl, 2008) were first used to compare susceptibility to ingroup (treatment factor 1) and outgroup influence (treatment factor 2) in judgments within the groups (EG, CG1, CG2; see Figure 1). To test Hypothesis 2 regarding group differences in susceptibility to ingroup influence, a second means-as-outcomes model was computed (Luke, 2004). This model again made it possible to consider participants’ intra-individual response variability to different experimental stimuli at level 1 and to analyze predictors on level 2 (Geiser, 2011). At level 2, it was tested whether differences between individuals in the dependent variable (*distance to the ingroup*; level 1; see Figure 1) were predicted by the level 2 predictor EG membership (CG1, CG2 with the EG as a reference category, dummy coded; Geiser, 2011).

## Results

### Preliminary Analyses

Intraclass correlations (cf. Geiser, 2011) were calculated to investigate the cluster structure and the relationship between the variance between the clusters (i.e., individuals) and the total variance of the dependent variables (i.e., polarization of social judgment, ingroup orientation). Using Mplus 8.1 software (Muthén and Muthén, 2017), the ICCs for polarization of social judgment (ρ_IC_ = 0.209) and ingroup orientation (ρ_IC_ = 0.504) were calculated using the unconditional model (i.e., the model without predictor variables). The results suggest that 20.9% of the variance in the polarization of social judgment was due to differences between individuals; for ingroup orientation 50.4% of the variance is due to differences between individuals.

Exploratory analyses were conducted to provide detailed information about some of the implemented models’ statistical assumptions. Levene’s tests for homogeneity of variances indicated that variances in the three groups (EG, CG1, and CG2) were not equal with respect to polarization of social judgment (*p* < 0.001) and ingroup orientation (*p* < 0.001). Furthermore, the residuals were tested for normality using a Kolmogorov–Smirnov test. For both polarization of social judgment and ingroup orientation, the residuals of the three groups were not normally distributed (*p* < 0.001). To identify outliers within the three groups regarding polarization of social judgment and ingroup orientation, boxplots were analyzed: mild outliers were characterized as being more than 1.5 times but less than three times the interquartile range, and extreme outliers were characterized as being more than three times the interquartile range. No outliers were found in polarization of social judgment in any of the three groups. Additionally, there were no outliers in the data collected from adolescents with ID and MA-matched children. In contrast, for ingroup orientation four adolescents without ID exhibited mild outliers on some single ratings, and two adolescents without ID exhibited extreme outliers on two ratings. No participant exhibited consistent outlier behavior across all items. Although these analyses reveal that some assumptions of the implemented models were violated, such deviations in samples of this size should not be a concern (Field, 2013). In addition, this situation is addressed by the use of a robust maximum likelihood estimator (MLR) in all models (cf. Muthén and Muthén, 2017). The MLR estimator is robust to non-normality and is applicable in the presence of heterogeneous variances (ibid.; Wooldridge, 2013).

### Hypothesis 1: Group Comparisons Regarding Polarization of Social Judgments

**FIGURE 2:**
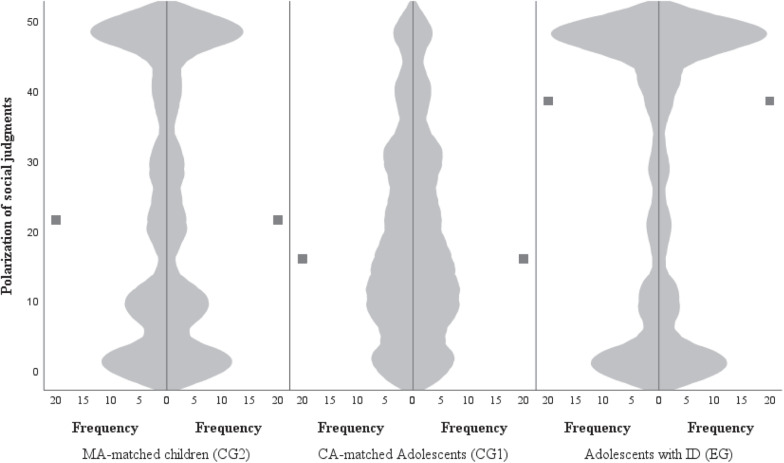
Polarization of social judgments within the three groups. Values close to 0 = ratings close to the center of the scale, values close to 50 = ratings close to the outer poles of the scale. Squares represent the medians. EG, experimental group comprising adolescents with intellectual disability (ID); CG1, comparison group 1 comprising chronological age-matched adolescents without ID; CG2, comparison group 2 comprising mental age-matched children.

Hypothesis 1 tested whether adolescents with ID make significantly more polarizing social judgments than typically developing adolescents. The descriptive results are listed in Table 2. Violin plots of polarization of social judgments provide additional descriptive insights into the distribution and medians of the data (see Figure 2). Medians are marked as two points lying at the same height next to the violin plots. While clustering toward 0 indicates a tendency in judgment toward the middle, clustering toward 50 indicates a polarizing judgment style. Roughly 50% of the ratings of adolescents with ID clustered in the outer quarters of the scale (*median* = 38.5). In addition to this tendency toward polarizing social judgment, a smaller clustering of ratings toward the middle of the scale is also evident. MA-matched children exhibit a similar distribution pattern (*median* = 21.5). Among adolescents without ID, about 50% of the ratings were located in the middle third of the scale (*median* = 16.0).

**TABLE 2:** Means and standard deviations of variables used in tests.

	**EG**		**CG1**		**CG2**	
	***M***	***SD***	***M***	***SD***	***M***	***SD***
Polarization	28.42	20.33	19.56	14.35	24.23	19.15
Distance to the ingroup	19.99	19.60	34.07	10.56	18.21	17.21
Distance to the outgroup	38.81	10.91	40.08	1.71	37.85	10.27

*EG, experimental group comprising adolescents with intellectual disability (ID); CG1, comparison group 1 comprising chronological age-matched adolescents without ID; CG2, comparison group 2 comprising age-matched children.*

The two-level means-as-outcomes model (Model 1) included *polarization of social judgment* (see Figure 1, T1) as a dependent variable; EG membership was specified on level 2 as a predictor (CG1 and CG2, with EG as a reference category, dummy coded; model fit: χ^2^(0) = 0.001, *p* < 0.001; RMSEA = 0.000; CFI = 1.000; TLI = 1.000). Consistent with Hypothesis 1, significantly more polarizing social judgments were found for the EG compared with the CG1 (*p* < 0.001; see Table 3). Hypothesis 1 was accordingly accepted. The standardized regression coefficient (β = −0.49; *p* < 0.001) indicated a moderate effect size (Cohen, 1988). No significant differences were found between the EG and CG2 (*p* = 0.101; see Table 3) regarding polarization of social judgments. In additional analysis that changed the reference category (CG1 as a dummy-coded reference category), significantly more polarizing judgments were found for CG2 compared with CG1 (*B* = 4.67, *SE* = 2.14, *p* = 0.029). The size of this effect was small using the standardized regression coefficient (β = 0.26; *p* = 0.021).

**TABLE 3:** Means-as-outcomes-models to predict group differences polarization (*N* = 102).

	***B***	***SE***	***z***	***p***
Intercept	28.42	1.88	15.12	<0.001
*Level 1: Experimental stimuli*				
*Level 2: Individuals*				
CA-matched adolescents (CG1)^a^	–8.86	2.26	–3.92	**<0.001**
MA-matched children (CG2)^a^	–4.19	2.56	–1.64	0.101
*Variances*				
Level 1 variance (within individuals)	269.37	18.26	14.76	<0.001
Level 2 residual variance (between individuals)	58.42	14.34	4.07	<0.001

*^a^ Reference category is adolescents with intellectual disability (EG).*

*Bold: effect of hypothesis test.*

*EG, experimental group comprising adolescents with intellectual disability (ID); CG1, comparison group 1 comprising chronological age-matched adolescents without ID; CG2, comparison group 2 comprising age-matched children.*

### Hypothesis 2: Group Comparisons Regarding Susceptibility to Ingroup Influence

**FIGURE 3:**
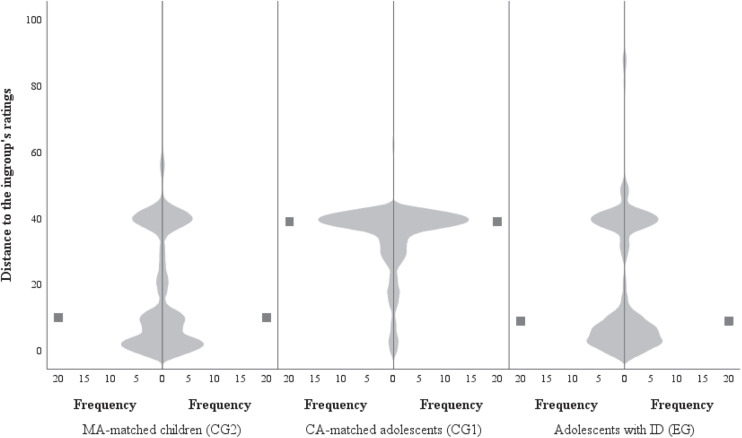
Distance of the participants’ ratings (T2) to ingroup ratings within the three groups. Values close to 0 = participant’s ratings (T2) close to ingroup ratings; values close to 40 = participant’s ratings (T2) close to the participant’s initial ratings (T1); values close to 100 = participant’s ratings (T2) in a large distance to the ingroup ratings. Squares represent the medians. EG, experimental group comprising adolescents with intellectual disability (ID); CG1, comparison group 1 comprising chronological age-matched adolescents without ID; CG2, comparison group 2 comprising younger age-matched children.

Hypothesis 2 tested whether adolescents with ID exhibit a significantly stronger susceptibility to ingroup influence compared with typically developing adolescents. Table 2 lists the respective descriptive results. Additional descriptive insight into the distribution and medians of the data is provided by violin plots (see Figure 3). The closer the distribution of the data is to 0, the stronger the ingroup orientation. Adolescents with ID (*median* = 9) and MA-matched children (*median* = 10) exhibited clustering of their judgments close to the ingroup. At the same time, a smaller clustering of ratings near the original judgment at T1 (T1 = 40) is visible for these two groups. Adolescents without ID exhibited 50% of judgments at 39 (median), which reveals that many judgments were made close to the original judgment at T1.

Preliminary analyses were conducted within the participant groups to compare any judgment shifts toward the ingroup with any judgment shifts toward the outgroup within the participant participant groups (EG, CG1, CG2, see Figure 1; T2; treatment factor 1 vs. treatment factor 2). This comparison allowed susceptibility to ingroup influence to be determined in contrast to susceptibility to outgroup influence. Wald tests were used to test the null hypothesis whether the difference between the variables of interest (*distance to the ingroup* vs. *distance to the outgroup*) was zero (Bühl, 2008). In all three participant groups a significantly stronger susceptibility to ingroup influence was found compared with susceptibility to outgroup influence (*p* < 0.001). In order to test Hypothesis 2, a means-as-outcomes model (Model 2) was conducted in accordance with the procedure of Luke (2004). The *distance to the ingroup* (see Figure 1; T2; treatment factor 1) was specified as a dependent variable and EG membership (CG1, CG2 with EG as a reference category, dummy coded) was included as a predictor at level 2 (model fit: χ^2^(0) = 0.000, *p* < 0.001; RMSEA = 0.000; CFI = 1.000; TLI = 1.000). As expected, participants in the EG exhibited a significantly stronger susceptibility to ingroup influence compared with participants in CG1 (*p* < 0.001; see Table 4), with a high effect size (β = 0.53; *p* < 0.001; Cohen, 1988). Hypothesis 2 was accordingly accepted. No significant differences in susceptibility to ingroup influence were found between the EG and CG2 (*p* = 0.596; see Table 4). Additional analyses that changed the reference category (CG1 as a dummy-coded reference category) revealed that participants in CG2 exhibited a significantly stronger susceptibility to ingroup influence compared with participants in CG1 (*B* = −15.85, *SE* = 2.55, *p* < 0.001). The size of this effect was high (β = −0.60; *p* < 0.001; Cohen, 1988).

**TABLE 4:** Means-as-outcomes-models to predict group differences in susceptibility to ingroup influence (*N* = 102).

	***B***	***SE***	***z***	***p***
Intercept	19.99	2.52	7.93	<0.001
*Level 1: Experimental stimuli*				
*Level 2: Individuals*				
CA-matched adolescents (CG1)^a^	14.07	2.82	4.99	**<0.001**
MA-matched children (CG2)^a^	–1.78	3.36	–0.53	0.596
*Variances*				
Level 1 variance (within individuals)	154.87	23.28	6.65	<0.001
Level 2 residual variance (between individuals)	107.44	17.90	6.00	<0.001

*^a^ Reference category is adolescents with intellectual disability (EG).*

*Bold: effect of hypothesis test.*

*EG, experimental group comprising adolescents with intellectual disability (ID); CG1, comparison group 1 comprising chronological age-matched adolescents without ID; CG2, comparison group 2 comprising age-matched children.*

### Further Analyses: Effect of Polarization on Susceptibility to Ingroup Influence

Since adolescents with and without ID differ in their polarization of social judgment and susceptibility to ingroup influence, it is of interest to analyze whether polarization of social judgment is correlated with ingroup orientation. No significant Spearman correlation was found between polarization and ingroup orientation in social judgment within the entire sample (*r*_*s*_ = 0.001; *p* = 0.988). When the individual groups (EG, CG1, and CG2) were analysed separately, the Spearman correlation between polarization of social judgment and ingroup orientation was also not significant (EG: *r*_*s*_ = 0.01, *p* = 0.951; CG1: *r*_*s*_ = 0.04, *p* = 0.682; CG2: *r*_*s*_ = 0.09, *p* = 0.296). These results suggest that these two constructs are not related.

Although no direct relationship between polarization of social judgment and ingroup orientation was found, polarization of social judgment could still have an effect on group differences in susceptibility to ingroup influence. Additional analyses were therefore conducted to determine whether the group difference in susceptibility to ingroup influence can be explained by polarization of social judgment. A means-as-outcomes model (cf. Luke, 2004; Geiser, 2011) with two levels was used to test group differences between the participant groups in their susceptibility to ingroup influence by controlling for polarization of social judgment on level 2 (i.e., differences in polarization between individuals). On level 1, the model considered participants’ intra-individual variability in *polarization of social judgment* and in *distance to the ingroup* for the different experimental stimuli (Geiser, 2011). On level 2, the *distance to the ingroup* (dependent variable) was predicted by group membership (CG1, CG2, with EG as a reference category; dummy coded) and *polarization of social judgment* on level 2 (model fit: (χ^2^(1) = 0.836, *p* = 0.361; RMSEA = 0.000; CFI = 1.000; TLI = 1.025). The EG exhibited a significantly stronger susceptibility to ingroup influence compared with participants in CG1 (*p* < 0.001; see Table 5). No significant differences in susceptibility to ingroup influence were found between the EG and CG2 (*p* = 0.715; see Table 5). Polarization of social judgment did not significantly predict susceptibility to ingroup influence (*p* = 0.134 see Table 5). This finding indicates that group differences between adolescents with and without ID in susceptibility to ingroup influence remained stable when additionally controlling for polarization of social judgment. This result suggests that different polarization tendencies cannot completely explain group differences in susceptibility to ingroup influence.

**TABLE 5:** Means-as-outcomes-model: Effect of polarization on susceptibility to ingroup influence (*N* = 102).

	***B***	***SE***	***z***	***p***
Intercept	9.62	7.19	1.34	0.181
*Level 1: Experimental stimuli*				
*Level 2: Individuals*				
CA-matched adolescents (CG1)^a^	16.77	3.19	5.25	<0.001
MA-matched children (CG2)^a^	–1.21	3.32	–0.37	0.715
*Polarization of social judgment*	0.39	0.26	1.50	0.135
*Variances*				
Level 1 variance (within individuals)	154.97	23.31	6.65	<0.001
Level 2 residual variance (between individuals)	99.47	18.57	5.36	<0.001

*^a^Reference category is adolescents with intellectual disability (EG).*

*EG, experimental group comprising adolescents with intellectual disability (ID); CG1, comparison group 1 comprising chronological age-matched adolescents without ID; CG2, comparison group 2 comprising age-matched children.*

## Discussion

This study examined the degree of polarization and susceptibility to ingroup influence in social judgment in adolescents with ID compared with chronological age-matched adolescents without ID and MA-matched children. The results indicate that adolescents with ID make more polarized social judgments and are more susceptible to ingroup influence than typically developing adolescents; they are comparable in these aspects to MA-matched children.

As expected in Hypothesis 1, adolescents with ID exhibited more polarizing social judgments than typically developing adolescents. The moderate size of this effect reveals that having an ID has an essential effect on polarization. However, additional factors besides ID may also play a role in polarization in social judgments. No differences in the polarization of social judgments between adolescents with ID and MA-matched children were found. These findings are consistent with the results of a previous study on polarization of social judgments in the field of social attractivity (i.e., coolness of adolescents; Egger et al., 2021) and with research on adolescents with ID regarding their stronger attribution of hostile characteristics to others compared with adolescents without ID (Van Nieuwenhuijzen et al., 2011; Van Rest et al., 2020). A tendency to extreme social judgments when judging strangers may be related to a reliance on biased mental representations (cf. Hiemstra et al., 2019). In adolescents with ID, mental representations may be biased due to specific social experiences (e.g., increased social conflicts; cf. Allen, 2000; Douma et al., 2014) and may lead to an increased attribution of negative or positive characteristics to others. However, it should be noted that the present experiment did not permit one to draw a conclusion about the specificity of the results to social judgments (there was no comparison condition using stimuli of a different content). Previous research has demonstrated that persons with ID and children without ID tend to exhibit more content-independent dichotomous response patterns when answering questions (cf. Chambers and Johnston, 2002; Kramer et al., 2009) compared with typically developing adolescents (Dekkers et al., 2017). This content-independent judgment style may be related to a simplistic thinking style and a preference for unambiguous information processing, which might manifest in a simple categorization of people into good and evil. In adolescents with ID, it may serve as a strategy for dealing with ambiguous judgment situations in order to achieve certainty (cf. Naemi et al., 2009; Acar-Burkay et al., 2014). In summary, adolescents with ID and MA-matched children exhibited more polarizing judgments compared with adolescents without ID; the exact underlying explanations cannot be specified based on the data available.

Consistent with Hypothesis 2, adolescents with ID exhibited a stronger susceptibility to ingroup influence in social judgment compared with typically developing adolescents, with a large effect size. No differences in susceptibility to ingroup influence were found between adolescents with ID and MA-matched children. These results are consistent with previous research demonstrating stronger openness to external influences among adolescents with ID and MA-matched children without ID compared with typically developing adolescents (cf. Bybee and Zigler, 1998). In the present study, stronger openness to the specific influence of the ingroup may be due to the fact that, unlike other groups, the ingroup often serves as a central reference in the acquisition of social knowledge (Hogg and Abrams, 1993). Furthermore, the current study extends the work of a previous study in which adolescents with ID and MA-matched children were found to be more susceptible to peer influence (without differentiation between in- and outgroup) than typically developing adolescents when judging the coolness of photographed adolescents (Egger et al., 2021). Within an intergroup context of peers (in- and outgroup), it may be that the ingroup is the primary source of social validation; agreement with the ingroup provides certainty and social consensus (cf. Shah et al., 1998). In the current study, adolescents with ID and MA-matched children may have used cues from the ingroup to maintain the simple dichotomous distinction between the ingroup and the outgroup and to situate themselves within this grouping structure. Taken together, adolescents with ID and MA-matched children exhibited stronger ingroup orientation compared with adolescents without ID. Varying explanations for this finding are possible, however, they could not be tested with the current data set.

Several studies have suggested that polarization of judgment and a strong preference for the ingroup (e.g., expressed as susceptibility to ingroup influence) may be due to a common underlying cognitive thinking style that consists of a preference for simplicity and a need to avoid ambiguity in information processing and judgment (Shah et al., 1998; Naemi et al., 2009; Acar-Burkay et al., 2014). According to this line of thinking, one would expect that these constructs are related. No correlation between polarization of social judgment and ingroup orientation was found in this study within the entire sample and within each group. Furthermore, the above line of thinking assumes that differences in polarization of social judgment can explain the effect of group differences in openness to ingroup influence. However, even after controlling for polarization of social judgment this study found that group differences in susceptibility to ingroup influence between adolescents with and without ID remained stable. This finding suggests that polarization of social judgment and susceptibility to ingroup influence must be explained by other motivational and cognitive factors (cf. Hogg and Abrams, 1993). Alternatively, one possible explanation for the polarization of social judgments among adolescents with ID may involve content-independent difficulties making subtle distinctions in judgment compared with typically developing adolescents (cf. Fang et al., 2011), which could contribute to more dichotomized judgments. The violin plot shown in Figure 2 might point in this direction: adolescents with ID and MA-matched children in particular use the poles and the middle of the scale to make judgments; the judgments of adolescents without ID are better distributed across the entire scale. An alternative possibility for increased susceptibility to ingroup influence in adolescents with ID may concern their desire not to be perceived as deviating from their peers (Snell et al., 2009), which could explain their heightened orientation toward the ingroup opinions. In summary, based on the analyses performed, the mechanisms activated during the polarization of social judgments and the ingroup orientation cannot be entirely explained.

### Limitations and Future Research Directions

To the best of the author’s knowledge, this study is the first to investigate the minimal group paradigm in adolescents with ID and matched comparison groups. Using a new experimental task, this research extends prior findings regarding the role of individual and contextual factors in social judgment making of adolescents with ID. However, it also has limitations.

In the computer-based task, the participants rated the hostility of virtually simulated people based on minimal information (i.e., external characteristics) provided. In this judgment situation, no explicit indications about each person’s actual hostility (e.g., as indicated by their facial expression) were available and the task was therefore ambiguous for all participants. One might suggest that no accurate answer was possible. However, the focus of this study was on polarizing social judgment style in social judgment situations with minimal information (e.g., clothing style), as is common in everyday first impressions (cf. Naumann et al., 2009; Over and Cook, 2018), rather than the correct social judgment of individuals based on external characteristics). This social judgment situation is consistent with Outerdirectedness research in which response behavior is examined when no obviously correct answer is possible (cf. Bybee and Zigler, 1998). The use of virtually simulated individuals, however, presents a possible limitation to the ecological validity of this study. While consistent results were found regarding polarization in social judgments of the coolness of photographed real people (Egger et al., 2021), no judgments of the hostility of real people were made with this sample. Due to the lack of complementary data regarding social judgments of hostility in real people, this study cannot make statements about the ecological validity of this judgment task. Following Schindler et al. (2017), such computer-based stimuli are valid for testing initial hypotheses about social judgment, but they require final validation with real photographed subjects. As a result, future studies should incorporate judgment tasks about hostility involving photographs of real people and additional observational data from social judgment making in the everyday lives of adolescents with ID. In addition, future studies should include judgments independent of social context to distinguish tendencies in social judgment making from tendencies in judgment making in other contexts.

In general, adolescents with ID and MA-matched children may have had more difficulties solving the task because it was ambiguous and no obviously correct answer was possible. This situation could have led to more polarizing judgments in adolescents with ID and MA-matched children. In addition, it is possible that adolescents with ID and MA-matched children had more difficulties using the rating scale for social judgment making compared with adolescents without ID. Although participants received training about using the scale, an additional alternative form of judgment making would be useful in future studies to determine the influence of the use of the scale on the results. The present study aimed to investigate the influence of an ingroup among adolescents with ID within an intergroup context. For this purpose, the opinions of the in- and outgroup (i.e., an outgroup with the same opinions as the participants and an ingroup whose opinions differed from those of the participants) were presented simultaneously. Additional follow-up research could add to the present findings by investigating outgroup influence and the interplay between in- and outgroup influence in intergroup contexts. Furthermore, it would be interesting to better understand group processes in adolescents with ID in different social contexts (e.g., among real peers or adults).

### Implications

Despite certain limitations, these study results have several implications. It was found that adolescents with ID tend to polarize judgments in situations where behavioral traits (e.g., the hostility of others) are derived only from external characteristics of the target persons. Judgment of personality traits based on external characteristics (e.g., clothing style) plays a significant role when forming first impressions (Naumann et al., 2009). In such social judgments, a simplifying judgment pattern made in black-and-white terms is related to increased susceptibility to prejudice and ideologies (Roets and Alain, 2011; Federico et al., 2013; Hodson and Dhont, 2015). Our study results suggest that this susceptibility may apply to adolescents with ID, thereby exposing them to social risks. For example, a highly polarized positive perception of others may hinder differentiation in social judgment making and increase susceptibility to social manipulation. A highly polarized negative perception of others, on the other hand, may contribute to one expressing negative judgments against these persons. Additional research is necessary to investigate the link between polarizing judgments in adolescents with ID and their susceptibility to prejudice and ideologies in terms of simplistic explanatory patterns.

The tendencies of adolescents with ID to make more general and content-independent polarizing judgments may be taken into account in everyday decision-making situations. For example, therapists and other professionals may help adolescents with ID establish familiarity with different response options *before* they have to make an important decision. Doing so may involve using visualized symbols such as a visual decision-making aid (cf. Dymond et al., 2010; Bailey et al., 2011). Such tools may facilitate access to different response options for adolescents with ID and therefore reduce ambiguity in their decision making.

Furthermore, adolescents with ID were found to be more susceptible to ingroup influence compared with typically developing adolescents, even without explicit social pressure from the ingroup. Given that the fictitious ingroup shared only a minimal distinguishing feature (i.e., the color of group membership) and no additional knowledge about other characteristics was introduced, it is remarkable that adolescents with ID strongly oriented toward this group. This increased susceptibility to social influence may contribute to heightened social risks in adolescents with ID: an unknown group may promise group membership if an adolescent with ID acts in a specific way, for example. However, an orientation toward an ingroup (in contrast with an outgroup) can also be considered a social learning opportunity for adolescents with ID. Accordingly, in certain contexts such as schools teachers may create affiliations by referring to similarities between an individual and positive social groups in order to promote a positive ingroup influence (cf. Diehl, 1990).

In summary, this study contributes to the still-scarce knowledge base of how adolescents with ID make social judgments. The insights gained point to the social vulnerabilities associated with ID and can contribute to providing perspectives for better support of adolescents with ID.

## Data Availability Statement

The raw data supporting the conclusions of this article will be made available by the authors, without undue reservation.

## Ethics Statement

The studies involving human participants were reviewed and approved by the institutional research committee of the Department of Special Needs Education at the University of Fribourg. Written informed consent to participate in this study was provided by the participants’ legal guardian/next of kin.

## Author Contributions

SE developed the experimental task in collaboration with Philipp Nicolay and Christian Huber from the University of Wuppertal (Germany) and Christoph Michael Müller (University of Fribourg, Switzerland), organized and took part in the recruitment of participants, data collection, conducted all data analyses, and wrote the manuscript.

## Conflict of Interest

The author declares that the research was conducted in the absence of any commercial or financial relationships that could be construed as a potential conflict of interest.

## Publisher’s Note

All claims expressed in this article are solely those of the authors and do not necessarily represent those of their affiliated organizations, or those of the publisher, the editors and the reviewers. Any product that may be evaluated in this article, or claim that may be made by its manufacturer, is not guaranteed or endorsed by the publisher.

## Footnotes

1
www.freepik.com
