# Supplementary material for: Susceptibility to Ingroup Influence in Adolescents With Intellectual Disability: A Minimal Group Experiment on Social Judgment Making
Source: Front Psychol. 2021 Aug 25;12:671910. doi: 10.3389/fpsyg.2021.671910 (PMC8423920; doi:10.3389/fpsyg.2021.671910)
Supplement: Supplementary file 1 [file Image_1.pdf]

Appendix

Figure 1

Depicted Virtually Simulated Persons of the Computer-Based Task

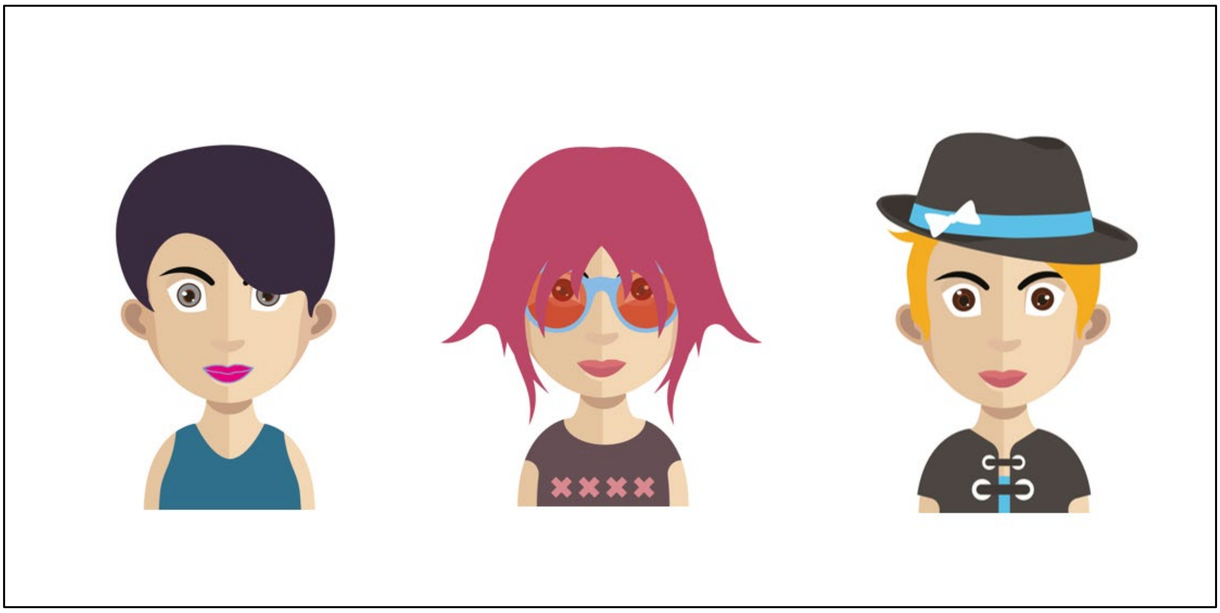

Note. Picture source: Freepik, 2020.
